# Clinicopathological Conference: 54-year-old with Facial Swelling for One Month

**DOI:** 10.5811/cpcem.2020.9.48439

**Published:** 2020-10-09

**Authors:** Justin Edwin Pile, Adam Dawson, Lynn Maxa

**Affiliations:** * Swedish Hospital, Part of NorthShore University HealthSystem, Department of Emergency Medicine, Chicago, Illinois; † Kingman Regional Medical Center, Department of Emergency Medicine, Kingman, Arizona

## Abstract

A 54-year-old female with facial swelling for one month who had repeatedly been treated for allergic reaction during multiple emergency department (ED) visits, presented to the ED for the same complaint of facial swelling. Maintaining a broad differential diagnosis was of critical importance to appropriately evaluating the patient and arriving at the correct conclusion for the etiology of the patient’s symptoms. Upon establishing the correct diagnosis, a multidisciplinary approach was used to intervene to provide early treatment without delay.

## CASE PRESENTATION (Resident Presentation)

A 54-year-old female with no past medical history presented to the emergency department (ED) of an urban community hospital with persistent facial swelling for more than one month despite five previous ED visits to diagnose and treat her condition. More than a month earlier, she had received a one-week course of clindamycin for a dental abscess, which she took just prior to the onset of facial swelling, redness, and itching. It was thought that these symptoms were due to an allergic reaction to the clindamycin. She was seen in an ED and was given diphenhydramine and prednisone with subjective improvement of the symptoms (follow-up visit one). Since that first ED visit for signs of an allergic reaction, she had four additional visits to different EDs with the same symptoms and was prescribed hydroxyzine during the second visit (follow-up visit two), loratadine during the third visit (follow-up visit three), and additional courses of prednisone in the last two of the five visits (follow-up visits four and five).

After the fifth follow-up visit for symptoms of a presumed allergic reaction, she presented for the same facial swelling this time to our ED. Her facial swelling was worse at night but did not fully resolve during the day. She denied any additional exacerbating or alleviating factors. Of note, the patient denied having a primary care physician or any physician who knew her medical history. At the previous ED visits, she stated that she thought labs had been drawn but was not certain and could not recall the specific dates she had visited each ED. She denied having had any imaging completed during the prior ED visits.

A review of systems was positive for facial swelling and negative for tongue swelling, oropharyngeal swelling, sore throat, dysphagia, chest discomfort, dyspnea, rash, pruritis, abdominal pain, nausea, vomiting, change in bowel or bladder habits, weight gain or loss, loss of appetite, and extremity swelling. She denied any other medical complaints. She denied any medical or surgical history, tobacco, alcohol, or illicit drug use, and stated that she lived with her husband.

**Image 1:**
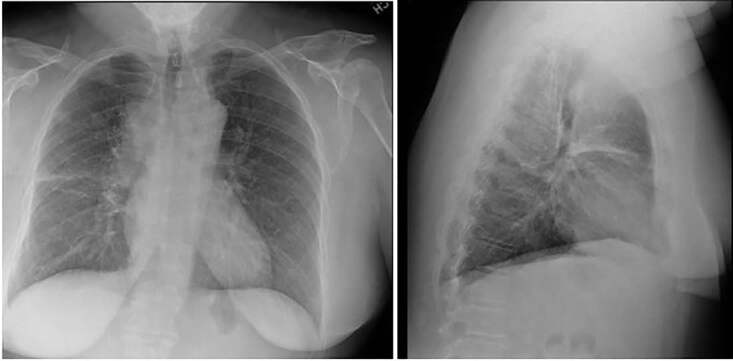
Posterior-anterior and lateral chest radiograph of a 54-year-old female with facial swelling.

**Image 2:**
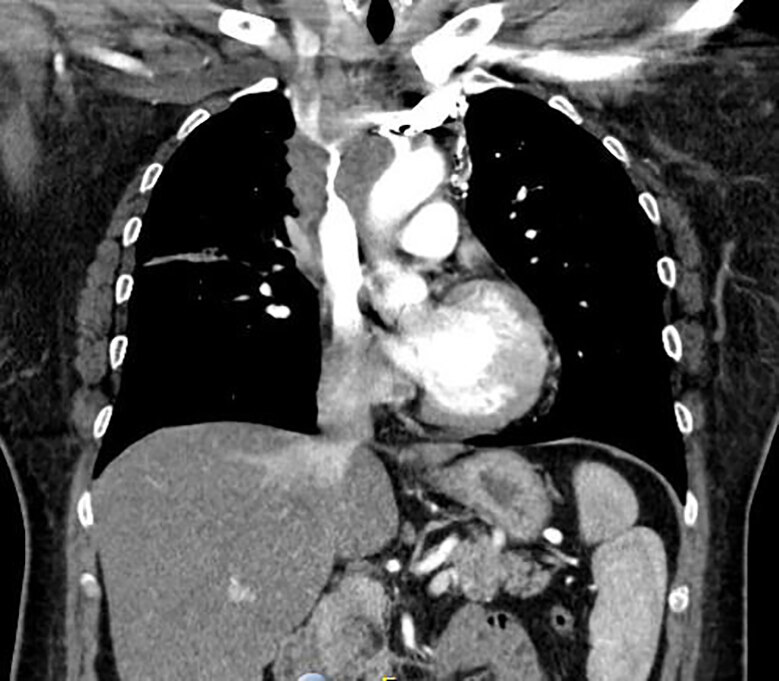
Computed tomography of the chest with intravenous contrast of a 54-year-old female with facial swelling.

Vital signs were as follows: temperature 98.1 degrees Fahrenheit; blood pressure 111/66 millimeters mercury; heart rate 82 beats per minute, respiratory rate 20 breaths per minute; and oxygen saturation 96% on room air. Physical examination revealed an obese, White female sitting awake and alert in no acute distress with significant diffuse swelling of her face, which was most prominent in the periorbital region without ecchymoses, erythema, or urticaria. Her conjunctivae were without erythema. Her pupils were equal, round, and reactive to light, and the extraocular motions were intact and painless. There were no other abnormal respiratory, cardiovascular, abdominal, or neurologic findings on examination. A complete blood count, basic metabolic panel, and chest radiograph (CXR) were ordered during the initial workup of the patient. Based on CXR findings (Image 1), a computed tomography (CT) of the chest was obtained (Image 2). The patient had an uneventful stay in the ED.

## CASE DISCUSSION (Attending Discussion)

The underlying cause of facial swelling can be difficult to determine. In the case of this 54-year-old female, she had undergone at least six evaluations prior to this particular presentation. The true first visit was a case of a dental abscess, and this started a pathway of continuing unsuccessful treatment for an allergic reaction to the antibiotic. The physician should consider a variety of possible etiologies for this swelling given the protracted course and unsuccessful treatments.

An allergic reaction is arguably the most likely noninfectious cause of the patient’s facial swelling in the absence of additional clues. Antihistamines and steroids were used and were likely appropriate. Perhaps these treatments did even help somewhat with the true underlying cause of the swelling. If the antibiotic was indeed the cause of the allergic reaction, then multiple rounds of treatment should not have been required without additional exposures to the allergen. This was the working diagnosis, which was presumptive and incorrect.

Was a dental infection entirely ruled out? Such infections can come in a variety of forms not the least of which would be an abscess. Ludwig’s angina is a progressive and dangerous bacterial infection that occurs in the submandibular space; the origin is dental. The physical exam showed no oral/mucosal swelling, redness or tenderness, and the face had a similar exam. The subjective responsiveness to the treatment of an allergy would also imply that infection is unlikely. In fact, quite possibly steroids would have worsened such an infection. Now that we have decided that we no longer think that this could be allergic or infectious, what else is there?

Individuals with autoimmune disorders who are being treated with steroids can have steroid-related facial swelling. Cushing’s syndrome can lead to increased weight gain including in the face. There are a variety of potential causes, but iatrogenic steroid use is a common one. Regardless, the fact remains that in this case the patient presumably had initial improvement with steroids, and the initial steroid use was insufficient to propagate such a syndrome.

Angioedema is yet another consideration for our patient’s symptoms. Given her age, if she had the hereditary variety of angioedema caused by a C1 esterase inhibitor deficiency, there likely would be a personal or family history of a previous event. Angioedema can also occur due to excessive bradykinin in some patients on angiotensin-converting enzyme inhibitors. In either scenario, the use of antihistamines and steroids is controversial and without formal consensus of indication.

A complete blood count and basis metabolic panel were ordered and noted for hypokalemia, mild leukocytosis, and normocytic anemia as seen in the Table, but were not otherwise suggestive of any paraneoplastic syndromes such as syndrome of inappropriate anti-diuretic hormone.

The truly pivotal clue provided is that the patient’s symptoms are in fact worse at night and better during the day. The reason that this makes sense is because there is an issue with the vasculature. The fact that it is worse at night has nothing to do with circadian rhythm and you should suspect superior vena cava (SVC) syndrome. If you see or diagnose this condition once it will forever be in your differential and the primary diagnosis in patients with that description. This syndrome is essentially the sequelae of the backed-up flow to the right heart through the SVC. It can of course lead to much more severe symptoms but does not have to. The treatment definitively would be to correct the underlying cause.

In evaluation, the plain film CXR shows mediastinal widening with a probable mass. The follow-up computed tomography (CT) of the chest indeed demonstrated a circumferential mass around the SVC with narrowing of the vessel. Specifically, there is a mediastinal mass measuring 8.4 × 6.1 × 5.3 centimeters (cm) with significant compression of the SVC as well as a 3-cm mass at base of neck right side compressing the jugular vein. In addition, there was attenuation of the upper/middle lobe segmental pulmonary arteries and bronchi with post-obstructive, right upper lobe pneumonitis, two pulmonary nodules representing metastatic disease in right upper lobe, and a mass in right lobe of liver compatible with metastatic disease.

Superior vena cava syndrome could most certainly have caused the timing of symptoms the patient experienced. When she was lying down sleeping at night, there was insufficient flow through the SVC into the right atrium. The resulting appearance would be edema of the face. When upright during the day, gravity assists in the flow through the narrow vessel to increase the return to the right heart. If the problem were more directly gravity dependent, such as in congestive heart failure, it would be more likely to see the edema in the lower extremities and the swelling would subside when the person is lying down.

Given that a more common cause of this syndrome is due to malignancy, the steroids that were provided in the patient’s care quite possibly were therapeutic in decreasing inflammation, increasing the luminal diameter, and allowing better flow to the heart. The underlying causes of this are usually slow-growing, and it can be easy to miss this diagnosis until the final realization despite multiple attempts.

## CLINICAL DIAGNOSIS

Superior vena cava syndrome due to a mediastinal mass.

## CASE OUTCOME

The management of this patient relied on finding a diagnosis that explained her persistent facial swelling despite treatment with steroids and antihistamines. The previous diagnosis of allergic reaction at prior ED visits was not the correct diagnosis for her clinical condition. Two diagnoses that seemed plausible were SVC syndrome and malignancy, both of which could contribute to the patient’s facial swelling. A CXR (Image 1) indicated a possible mass that warranted chest CT (Image 2), which established the diagnosis of SVC syndrome secondary to a mediastinal mass.

The patient was transferred to another facility with cardiothoracic surgeons and pulmonologists. The patient was eventually diagnosed with small cell lung carcinoma with metastases to the mediastinum, liver, and supraclavicular lymph nodes. Upon follow-up of the patient, palliative surgery was considered for her mediastinal mass and radiation considered for the metastases. The final decision by the patient for treatment is not known.

## RESIDENT DISCUSSION

Superior vena cava syndrome is a collection of clinical signs and symptoms resulting from thrombus formation or tumor infiltration of the vessel wall causing either partial or complete obstruction of blood flow through the SVC.^1^ When the SVC is obstructed, there is retrograde flow to the left and right innominate (brachiocephalic) veins, which join to form the SVC.^1^ Thus, blood is unable to return to the heart and backs up into the head, neck, upper extremities and torso.^1^

This syndrome occurs in approximately 15,000 people in the United States annually.^2^ Originally described as being secondary to infection (tuberculosis or syphilitic aortic aneurysm), now it is now generally considered to result from cancer or thrombotic events.^2^ Most cases of SVC syndrome are associated with advanced malignant diseases that cause invasion of the venous intima or extrinsic mass effect.^2^ Common causes include lung, breast, and mediastinal neoplasms, with adenocarcinoma of the lung the most common cause.^2^ Non-Hodgkin’s lymphoma and then metastatic tumors are the second most common etiologies of SVC syndrome, followed by benign or nonmalignant causes, which comprise at least 40% of cases.^1^ Pacemaker wires and semipermanent intravascular catheters used for hemodialysis, long-term antibiotics, or chemotherapy are causes of iatrogenic thrombus formation and SVC stenosis.^1^

Gradual compression of the SVC leads to edema and retrograde flow. In thrombotic cases, this may result in an abrupt onset of symptoms.^3^ Signs and symptoms include swelling of the face, head, neck, breast, cough, dyspnea, distended neck veins, orthopnea, and conjunctival suffusion; collateral circulation leads to distension of superficial veins in the chest wall.^3^ Other less common symptoms of SVC syndrome include stridor, hoarseness, dysphagia, pleural effusion, head plethora, headache, nausea, lightheadedness, syncope, change in vision, altered mental status, upper body edema, cyanosis, papilledema, stupor, and coma.^1^ Some rare but serious clinical consequences reported in SVC syndrome include cerebral edema and upper respiratory compromise secondary to edema of larynx and pharynx.^1^

A physical exam maneuver associated with SVC syndrome is Pemberton’s sign, which is positive when bilateral arm elevation causes facial plethora.^4^ It has been attributed to a “cork effect” resulting from the thyroid obstructing the thoracic inlet, thereby increasing the pressure on the venous system.^4^ Pemberton’s maneuver is a clinical test for latent SVC syndrome caused by a substernal mass.^5^ If there is high clinical suspicion, imaging of the upper body and vasculature should be completed including ultrasound of the jugular, subclavian, and innominate veins to assess for thrombus, CXR, CT of the chest, or magnetic resonance imaging to assess for severity of the SVC obstruction. Venography is the gold standard for diagnosing venous obstruction.^1^ Multidisciplinary cooperation among radiation and medical oncologists and interventional radiologists is needed to provide an early treatment.^6^ Treatment involves chemotherapy and radiation for underlying cancer to reduce the degree of obstruction. Dilation and stenting as well as bypass of the SVC may be performed. Adjunctive therapies may include diuretics and corticosteroids.^1^

## FINAL DIAGNOSIS

Superior vena cava syndrome

## KEY TEACHING POINTS

- Superior vena cava syndrome is a collection of clinical signs and symptoms resulting from either partial or complete obstruction of blood flow through the SVC.
- SVC syndrome is most commonly due to cancer or thrombotic events.
- Upper body and facial swelling, cough, and shortness of breath are common symptoms.
- The swelling is commonly worse when the patient is lying flat, which is more frequently at night.
- Treatment can involve chemotherapy and radiation; dilatation, stenting, and bypass of the SVC; and diuretics and steroids.
